# Isolation of *Staphylococcus pseudintermedius* in Immunocompromised Patients from a Single Center in Spain: A Zoonotic Pathogen from Companion Animals

**DOI:** 10.3390/microorganisms12081695

**Published:** 2024-08-16

**Authors:** Joaquim Viñes, Miguel Ángel Verdejo, Laura Horvath, Andrea Vergara, Jordi Vila, Olga Francino, Laura Morata, Mateu Espasa, Climent Casals-Pascual, Àlex Soriano, Cristina Pitart

**Affiliations:** 1 Servei de Microbiologia i Parasitologia-CDB, Hospital Clínic de Barcelona, 08036 Barcelona, Spain; 2 Institut de Salut Global (ISGlobal), 08036 Barcelona, Spain; 3 Servei Veterinari de Genètica Molecular (SVGM), Facultat de Veterinària, Universitat Autònoma de Barcelona, 08193 Bellaterra, Spain; 4 Departament de Malalties Infeccioses, Hospital Clínic de Barcelona, 08036 Barcelona, Spain; 5 Institut d’Investigacions Biomèdiques August Pi i Sunyer (IDIBAPS), Centre Esther Koplowitz (CEK), 08036 Barcelona, Spain; 6 Departament de Fonaments Clínics, Facultat de Medicina i Ciències de la Salut, Universitat de Barcelona, 08007 Barcelona, Spain; 7 CIBER Enfermedades Infecciosas (CIBERINFEC), 28007 Madrid, Spain; 8 Nano1Health S.L. (N1H), Edifici EUREKA, Parc de Recerca UAB, 08193 Bellaterra, Spain

**Keywords:** *Staphylococcus pseudintermedius*, ST551, MRSP, MDR, zoonosis, SSTI, whole-genome sequencing, catheter-related infection, nanopore, one health

## Abstract

*Staphylococcus pseudintermedius*, a commensal opportunistic bacterium predominantly residing in the skin of companion animals, particularly dogs, has the potential to induce skin and soft tissue infections in pets, and zoonotic infections, including catheter-related complications. This study documents four cases of *S. pseudintermedius* infection or colonization in patients who had close contact with dogs or cats. Identification of the bacterial species was performed using MALDI-TOF mass spectrometry, and antibiotic susceptibility was determined using microdilution assay. DNA was sequenced using Nanopore technology followed by in silico analysis. Three isolates were multidrug resistant, including resistance to methicillin, with one belonging to the prevalent European lineage ST551, and the other two were attributed to a novel multilocus sequence type, ST2672. The remaining isolate was attributed to the novel multilocus sequence type ST2673 and was methicillin susceptible. All four isolates exhibited an array of virulence factors that contributed to colonization, damage to host immune cells, and biofilm formation. All the ST551 isolates included in the comparative analysis displayed clonality within the European continent. The importance of describing zoonotic infections associated with *S. pseudintermedius* resides in the scarcity of available scientific literature, further accentuated by its heightened resistance profile and potential complications, particularly in the context of catheter-related infections.

## 1. Introduction

Over the years, there has been a tendency to misclassify coagulase-positive (CoPS) *Staphylococcus* isolates, with most being identified as *Staphylococcus aureus*. In 1976, Hájek identified a novel CoPS, *Staphylococcus intermedius*, from pigeons, dogs, minks, and horses, noting its distinctive biochemical properties [1]. Over the following years, various *Staphylococcus* species were added to the *S. intermedius* group (SIG), including *Staphylococcus pseudintermedius* in 2005 [2]. Despite recognizing *S. pseudintermedius* as a species, classification challenges persist due to similar biochemical properties [3].

*S. pseudintermedius* is a commensal opportunistic bacterium found in approximately 90% of dogs and also in other companion animals like cats, inhabiting skin and mucocutaneous sites such as the nose, mouth, groin, and perineum [4,5]. The transition from commensal to opportunistic behavior may be influenced by skin lesions, compromised immunity, antibiotic treatment, and external factors, often leading to skin and soft tissue infections, otitis externa, urinary tract infections, and postoperative infections [4,6,7]. Notably, *S. pseudintermedius* often harbors methicillin resistance (MRSP), mainly due to the *mecA* gene within the *Staphylococcus* chromosome cassette *mec* (SCC*mec*) [8,9,10]. MRSP strains typically exhibit multidrug resistance (MDR) [11], posing treatment challenges and raising concerns about zoonotic potential [12].

A widely used method for classifying and establishing clonal associations of *S. pseudintermedius* is multilocus sequence typing (MLST). In Europe, the predominant sequence type is ST71, which exhibits high antibiotic resistance. However, other MLST are gaining relevance, such as ST496 in France, known for having resistance to a broad range of antibiotics, as well as ST258 and ST551 [4,9,13,14,15,16].

Unlike in dogs, colonization by *S. pseudintermedius* in humans is usually sporadic or considered as contamination [17,18,19,20,21], with immunocompromised patients [22,23] and veterinary dermatologists [24] being the most likely to be colonized by this microorganism. Even though a mortality rate for *S. pseudintermedius* infections in humans has not yet been determined, there have been fatal cases involving this organism. Balkhed et al. described a case of a 77-year-old male with fatal bacteremia, most likely caused by *S. pseudintermedius*, possibly transmitted by one of the dogs with which he shared a household [25]. In their 2023 publication, Moses et al. [3] summarized reports of *S. pseudintermedius* colonization and infection in humans from 2006 to 2022, including 48 studies with only three from Spain [26,27,28]. This study describes four cases of *S. pseudintermedius* infections in immunocompromised patients who had been in contact with dogs and/or cats. It provides a comprehensive analysis of these strains, evaluating their antibiotic resistance profiles both phenotypically and genotypically, and assessing the presence of virulence factors.

## 2. Materials and Methods

### 2.1. Patients and Isolates

From February to June 2023, four individuals with diverse comorbidities attended at the Hospital Clínic de Barcelona and *S. pseudintermedius* was isolated from skin (infection or colonization).

### 2.2. Strain Identification and Antimicrobial Susceptibility

Routine species identification was performed using MALDI-TOF MS (matrix-assisted laser desorption/ionization time-of-flight mass spectrometry) (Bruker Daltonics GmbH & Co. KG, Bremen, Germany). Antimicrobial susceptibility was assessed via automated microdilution for all antibiotics (oxacillin, erythromycin, clindamycin, gentamicin, vancomycin, rifampicin, levofloxacin, linezolid, trimethoprim-sulfamethoxazole, tetracycline) (Phoenix M50, Becton–Dickinson, New York, NY, USA), and oxacillin disk-diffusion was used for methicillin resistance screening, as well as rifampicin, following EUCAST recommendations of medium, inoculum, incubation time, and quality control reference strain (BD BBL Sensi-Disc, Becton–Dickinson, New York, NY, USA), following the interpretative breakpoints of the European Committee on Antimicrobial Susceptibility Testing (EUCAST v13.0).

### 2.3. DNA Extraction and Sequencing

DNA extraction was conducted using the ZymoBIOMICS^TM^ DNA miniprep Kit (Zymo Research, Irvine, CA, USA), following the manufacturer’s protocol. Subsequently, the assessment of DNA quality and quantity was performed using the NanoDrop^TM^ 2000 Spectrophotometer and Quantus^TM^ Fluorometer with the QuantiFluor^®^ dsDNA System (Promega, Madison, WI, USA), respectively.

Oxford Nanopore Technologies (ONT) libraries were prepared with approximately 400 ng of DNA using the Rapid Barcoding Sequencing kit (SQK-RBK004, ONT, Oxford, UK). Sequencing was carried out on a MinION FLO-MIN106 v9.1.4 flow cell (ONT, Oxford, UK) and the MinION Mk1C device (ONT, Oxford, UK). Live basecalling was chosen, and reads with a quality score < 8 and ≤200 bp were excluded from further analysis.

### 2.4. Genome Assembly and Analysis

Genome assembly was performed with Flye v2.9.2 [29] and polished with Medaka v.1.7.2 [30]. Completeness was assessed with CheckM v1.2.2 [31] and BUSCO v5.3.2 [32].

MLST were determined using PubMLST [33] and MLST 2.0 from the Center for Genomic Epidemiology (CGE) [34]. Three isolates presented two previously undescribed sequence types (23-298 and 23-583 shared the same new allele combination). The seven-allele combination for these isolates was submitted to the *S. pseudintermedius* PubMLST typing database, and a novel sequence type was assigned to both combinations.

Since isolate 23-150 belonged to the MLST ST551, a search for ST551 genomes was conducted within the NCBI Nucleotide database using the query “pseudintermedius AND ST551”, from which we retrieved 19 genomes (1 from *Felis catus*, 13 from *Canis lupus familiaris*, and 5 from environmental samples). Representative genomes corresponding to ST159 and ST498, the nearest sequence types to the newly identified ST2673 in this study, were also sought, retrieving one and two genomes isolated from dogs, respectively. The terms “pseudintermedius and “Homo sapiens”” were concurrently used to retrieve the first 23 genomes of *S. pseudintermedius* isolated from human cases. Consequently, our dataset encompassed a total of 50 genomes, including the reference genome (CP065921.1) and the isolates from our study (refer to Supplementary Table S1 for details of all the genomes from NCBI). Phylogeny was inferred by calling single nucleotide polymorphisms (SNPs) using CSIPhylogeny [35] v1.4. The Newick file obtained was visualized using FigTree v1.4.4 (available at http://tree.bio.ed.ac.uk/software/figtree/, accessed on 15 January 2024). Average nucleotide identity (ANI) against the 46 downloaded genomes was calculated with FastANI [36].

The presence of antibiotic resistance genes (ARGs) was assessed with Abricate v1.0.1 [37] alongside CARD [38] and NCBI databases. Mutations in *gyrA* and *parC* genes for fluoroquinolone resistance were described by aligning the genes with the *S. pseudintermedius* reference genome genes using BioEdit v7.2.5 [39]. SCC*mec*Finder 1.2 from CGE (available at https://cge.food.dtu.dk/services/SCCmecFinder/, accessed on 15 January 2024) was used to identify the SCC*mec* type within the genomes included in this study, and for elements not listed in the software’s database, a BLAST [40] was performed to find the best match. The presence of virulence factors was assessed with Abricate v1.0.1 with a previously described custom database (SPVFDB) which includes 58 genes [41]. Genome annotation was performed with Prokka [42] v1.14.6 and visualized with SnapGene Viewer v5.2.4 (from Insightful Science, San Diego, CA, USA; available at https://www.snapgene.com/, accessed on 15 January 2024). SCC*mec* synteny analysis was performed using EasyFig v2.2.5 [43].

## 3. Results

### 3.1. Medical History and Isolates

Retrospectively, all skin and soft tissue samples with *S. pseudintermedius* isolated from January to July 2023 in the Clinical Microbiology department of the Hospital Clínic de Barcelona were included (Table 1).

### 3.2. Antibiotic Susceptibility Profile

Susceptibility testing (Table 2) showed that ST511 (23-150) and ST2672 (23-298 and 23-583) isolates were MRSP and MDR, resistant to at least six antibiotic families: β-lactams, macrolides, lincosamides, fluoroquinolones, trimethoprim-sulfamethoxazole, and tetracyclines. Isolate 23-150 was only susceptible to vancomycin, rifampicin, and linezolid, while ST2672 isolates were susceptible to gentamicin, vancomycin, rifampicin, and linezolid. Isolate 23-472 (ST2673) was susceptible to all tested antibiotics.

Supplementary Table S2 shows antibiotic susceptibility from additional studies of ST551 isolates. While not all studies examined identical antibiotics, concordant antibiotics showed similar susceptibility patterns to isolate 23-150.

### 3.3. Antibiotic Resistance Genotype

In silico analysis identified 11 ARGs, and two and one mutations in the *gyrA* and *parC* genes, respectively (Table 2).

Isolate 23-472 only had the *blaZ* gene. ST2672 isolates (23-298 and 23-583) had seven ARGs and two mutations. The *mecA* and *blaZ* genes were linked to β-lactam resistance, covering oxacillin, with no SCC*mec* type found using SCC*mec*Finder. It showed high homology to *S. pseudintermedius* strain 57395 (GenBank: HE984157.2), classifying it as ΨSCC*mec*_57395_. The *tet(M)* and *tet(L)* genes conferred tetracycline resistance, while *aac(6′)-Ie-aph(2″)-Ia* was linked to aminoglycoside resistance. The presence of *erm(B)* indicated resistance to macrolides and lincosamides (erythromycin and clindamycin), and *dfrG* was responsible for trimethoprim resistance, as seen with trimethoprim-sulfamethoxazole. Mutations in *gyrA* S84L and *parC* S80I were linked to fluoroquinolone resistance (e.g., levofloxacin) [44].

Isolate 23-150 (ST551) had more ARGs than the ST2672 isolates. It contained the *blaZ* gene, the *mecA* gene within an SCC*mec* type Vc, the *aac(6′)-Ie-aph(2″)-Ia* gene, the *erm(B)* gene, the *dfrG* gene, and the *tet(M)* gene. It lacked the *tet(L)* gene but had the *tet(K)* gene. Additionally, it had the *aph(3′)-IIIa* and *ant(6)-Ia* genes for aminoglycoside resistance and the *sat4* gene for streptothricin resistance. Compared to other ST551 isolates (Supplementary Table S3), they showed a nearly identical ARG profile; only one isolate lacked *ant(6)-Ia* and e*rm(B)*.

### 3.4. Antibiotic Resistance Genes’ Genomic Context

**Figure 1:**
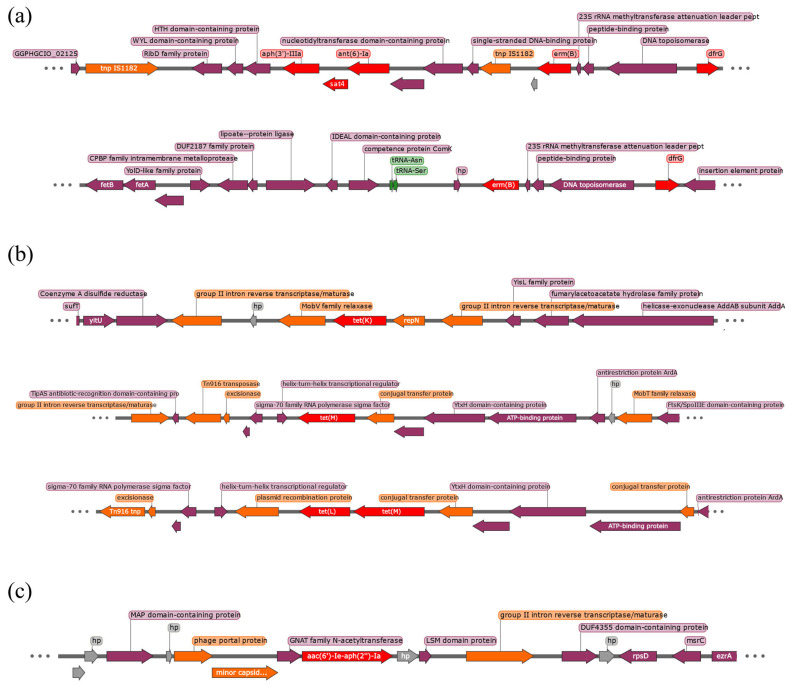
Genomic context of the ARGs. (**a**) genomic context for *aph(3′)-IIIa*, *sat4*, *ant(6)-Ia*, *erm(B)*, and *dfrG*. The first line corresponds to isolate 23-150, and the second to isolate 23-298; (**b**) genomic context for *tet(K)*, *tet(L)*, and *tet(M)* genes. The first two lines correspond to isolate 23-150 and the third one to isolate 23-298; (**c**) genomic context for *aac(6′)-Ie-aph(2″)-Ia* gene. This line corresponds to isolate 23-150, but ST2672 isolates present the same genomic context with the exception of the “group II intron reverse transcriptase/maturase”.

The genomic context of all ARGs, excluding *blaZ*, was examined. Figure 1a shows a distinct cluster of five genes (*aph(3′)-IIIa*, *sat4*, *ant(6)-Ia*, *erm(B)*, and *dfrG*) in isolate 23-150. The first three genes are flanked by insertion sequence elements. In contrast, ST2672 isolates (23-298 and 23-583) lack these three genes, with *erm(B)* adjacent to two tRNA genes.

For tetracycline resistance (Figure 1b), isolate 23-150 had both *tet(K)* and *tet(M)* genes. The *tet(K)* gene was located between plasmid-related genes, suggesting possible plasmid integration into the chromosome. The *tet(M)* gene was surrounded by elements that might aid in its mobilization. In ST2672 isolates, the *tet(L)* and *tet(M)* genes were adjacent, indicating potential plasmid integration of the *tet(L)* gene.

The *aac(6′)-Ie-aph(2″)-Ia* gene was found in isolate 23-150 and ST2672 isolates (Figure 1c), sharing a similar genomic context, differing only by the presence of a “group II intron reverse transcriptase/maturase” element. Downstream of this gene, genes encoding phage elements were annotated.

**Figure 2:**
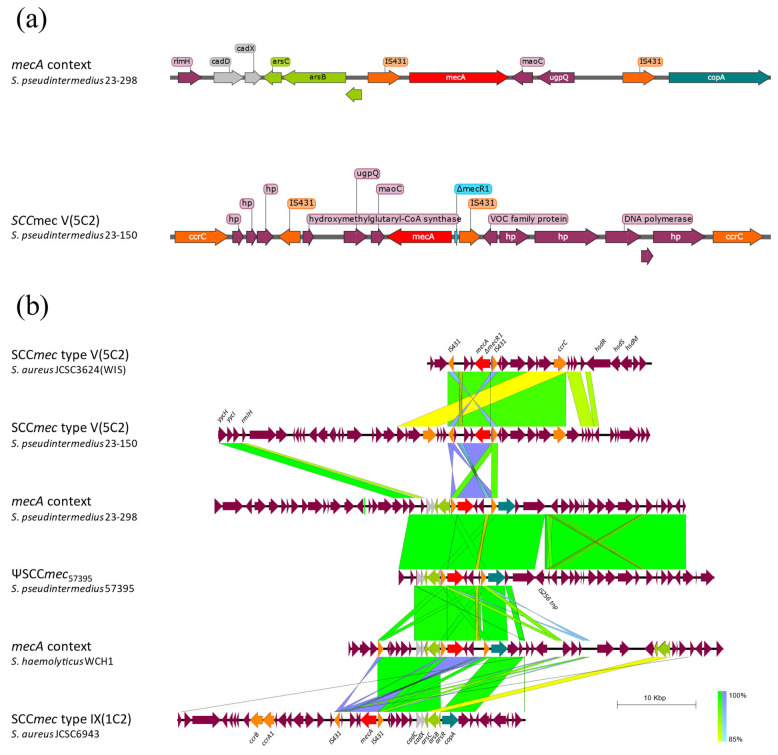
Genomic context of *mecA*. (**a**) SnapGene Viewer visualization of the genomic context of *mecA* in isolates 23-150 and 23-298. (**b**) EasyFig comparison of the genomic context of *mecA* in isolates 23-150 and 23-298 with SCC*mec* type V(5C2) from *S. aureus* JCSC3624(WIS) (GenBank: AB121219.1), ΨSCC*mec*_57395_ pseudocassette from *S. pseudintermedius* strain 57395 (GenBank: HE984157.2), *mecA* context from *S. haemolyticus* WCH1 (GenBank: JQ764731.1), and SCC*mec* type IX(1C2) from *S. aureus* JCSC6943 (GenBank: AB505628.1). In red, *mecA* gene; in orange, elements responsible for mobilization and reorganization of the cassette; in light blue, ∆*mecR1*; in green, gray, and teal, genes responsible for arsenic, cadmium, and copper resistance, respectively. Homology gradients range from green (100%) to yellow (85%), and violet to light blue (85%) for inverted regions.

The basic structure of SCC*mec* includes three main components: the *ccr* gene complex (coding for recombinases that mobilize the cassette); the *mec* gene complex (harboring the *mecA* gene, regulator genes, and insertion sequences like IS431); and a J region containing non-essential elements. Isolate 23-150 had SCC*mec* V(5C2) according to SCC*mec*Finder, while isolates 23-298 and 23-583 had a pseudocassette lacking the *ccr* gene complex, denoted as ΨSCC*mec*_57395_ (Figure 2a).

The SCC*mec* V(5C2) in isolate 23-150 showed 100% homology with the SCC*mec* V(5C2) sequence from *S. aureus* JCSC3624 (WIS) (AB121219.1) (Figure 2a, first and second lines). It included two copies of IS431 in opposite directions flanking the *mec* gene complex, with one copy truncating the *mecR1* regulatory gene, and two copies of the recombinase *ccrC*. In contrast, isolates 23-298 and 23-583 lacked the *ccr* gene complex, with the *mecA* gene and one copy of IS431 inverted (Figure 2a, second and third lines).

Isolates 23-298 and 23-583 had a genomic configuration for the *mecA* gene lacking the *ccr* gene complex, showing 100% homology with the ΨSCC*mec*_57395_ element identified in *S. pseudintermedius* strain 57395 by Perreten et al. [45], except for the absence of an IS256 *tnp* gene in our isolates (Figure 2a, third and fourth lines). This pseudocassette includes two IS431 copies in the same orientation, as well as resistance genes for cadmium (*cadC* and *cadX*), arsenic (*arsB*, *arsC*, and *arsR*), and copper (*copA*), situated adjacent to the IS431 copies. A similar configuration with metal-resistance genes has been observed in other staphylococci, such as *Staphylococcus haemolyticus*, as described by Zong [46] (Figure 2a, fifth line). Additionally, other SCC*mec* types have been reported to contain the same metal-resistance genes but in a different configuration, like in SCC*mec* IX(1C2), where all the genes are clustered (Figure 2a, sixth line).

### 3.5. Virulence Factors

A total of 58 virulence factor (VF) genes were examined, covering categories like surface proteins, regulators, proteases, toxins, signal transducers, exoenzymes, and modulins. The study’s isolates shared 45 VF genes, with isolate 23-150 missing the *nanB* gene responsible for sialidase production (Supplementary Table S4). Notable findings included 18 surface protein genes, highlighting the *ica* locus crucial for biofilm formation [47,48], and 10 regulation-related genes, including the *agr* locus for quorum sensing and VF expression [49]. Seven toxin genes were identified, including *lukF-*I and *lukS-*I (leukocidin), and exfoliative toxins such as *siet* and *speta* [6,49]. Additionally, all isolates had four *psm* genes involved in biofilm stabilization [50].

An analysis of 46 genomes from NCBI revealed 52 VF genes, with several unique genes compared to the study’s isolates. Notably, genes encoding surface proteins (*spsF*, *spsO*, *spsP*, and *spsQ*) and toxins (*exi*, *expB*, and *seccanine*) were present in a minority of the genomes, indicating variability in virulence factors among different strains.

### 3.6. Phylogenetic and ANI Analysis

**Figure 3:**
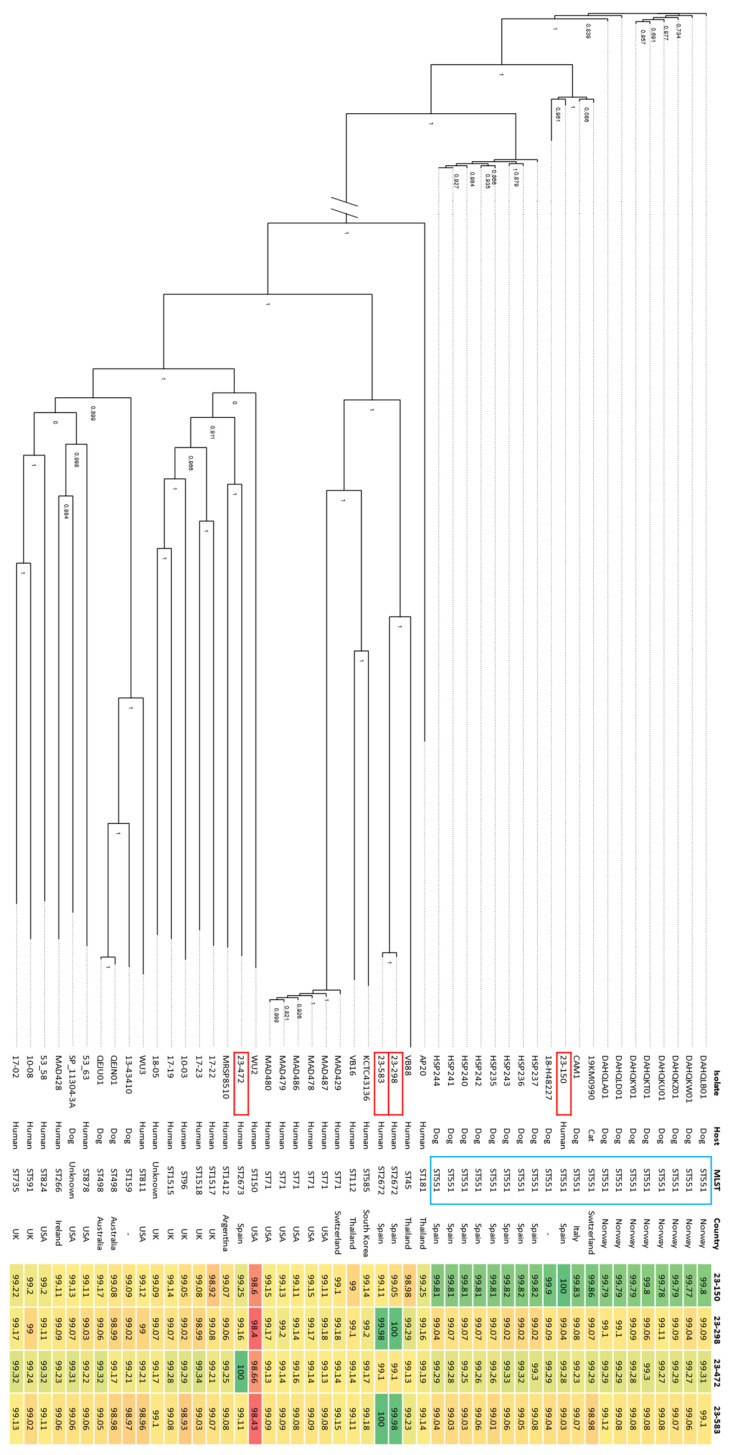
Phylogenetic tree and ANI. We performed phylogenetic analysis with all the ST551 *S. pseudintermedius* genomes available at NCBI nucleotide database, and other *S. pseudintermedius* isolated from *Homo sapiens*. ANI results are expressed as percentages and displayed in a heat map, with green boxes indicating the highest identities and red boxes indicating the lowest identities. Information regarding host disease, isolation source, and sequencing technology used can be found in Supplementary Table S1. MLST, multilocus sequence type; UK, United Kingdom; USA, United States of America.

Phylogenetic analysis based on SNPs and ANI analysis compared the isolates’ genomes with genomes from NCBI, including ST551 genomes and other human isolates (Figure 3). All ST551 genomes from NCBI were of canine origin, except for one from a cat, and were distributed across countries like Spain, Italy, Norway, and Switzerland.

Isolate 23-150 (ST551) showed high ANI identity with other ST551 isolates, ranging from 99.79% to 99.9%. All ST551 genomes formed a distinct cluster within the phylogenetic tree, with isolate 23-150 closely related to isolate 18-H48227, sharing a 99.9% ANI. Isolates 23-298 and 23-583 showed the highest identity similarity with a ST45 isolate from Thailand (VB88), both in ANI identity and phylogenetic placement. Isolate 23-472 had its highest ANI similarity with an ST1518 isolate from the UK (17–23) but clustered more closely with isolate MRSP8510 ST1412 from Argentina in the phylogenetic tree, both from human cases. The four study isolates had the least homology with the ST150 isolate from the USA, with ANI ranging from 98.40% to 98.66%.

## 4. Discussion

*S. pseudintermedius* is part of the canine skin microbiota and can cause zoonotic infections. Historically, it has been misidentified as other *Staphylococcus* species, such as *S. aureus* and *S. intermedius* [51], due to, for example, limitations in API databases lacking comprehensive representation of zoonotic pathogens. Moreover, the delayed and poor response of *S. pseudintermedius* to slide coagulase and commercial latex agglutination tests has led to its erroneous categorization as coagulase-negative *Staphylococcus* [3]. The introduction of MALDI-TOF has significantly reduced these misidentifications [52].

This study documents four cases of *S. pseudintermedius* infection or colonization from February to June 2023 at the Hospital Clínic de Barcelona, Spain. Isolate 23-150 belonged to ST551, while isolates 23-298 and 23-583 were ST2672, and isolate 23-472 was ST2673 (both being novel MLSTs). Both ST551 and ST2672 isolates were MRSP, featuring SCC*mec* type V and ΨSCC*mec*_57395_, respectively. The ΨSCC*mec*_57395_ element was originally characterized by Perreten et al. in 2013 [45] and is associated with clonal complex 45 (CC45), including ST45 [45,53]. In the phylogenetic tree, the ST2672 isolates were closely related to isolate VB88, an ST45 strain from a human source known to harbor this pseudocassette [54]. Isolate 23-472 was susceptible to all tested antibiotics, whereas both ST551 and ST2672 isolates exhibited MDR in both genotype and phenotype.

ST71 *S. pseudintermedius* is the predominant lineage in Europe, known for its MDR profile, including methicillin resistance [4,13]. However, other MLSTs are potentially supplanting ST71. In France, ST496 has emerged, showing resistance to all veterinary-licensed antibiotics [9], and the spread of ST258 is notable, potentially displacing ST71. This last lineage of *S. pseudintermedius* exhibits higher antibiotic susceptibility, reduced biofilm formation, and lower adherence to corneocytes than other lineages [9,16,55,56]. A third MLST, ST551, is distributed throughout Europe. This MLST has been observed in Spain [41,57], Portugal [58,59], Italy [60], Sweden [61], Norway [18], Slovenia [15], Poland [62,63], and Switzerland [64]. ST551 isolates have been recovered from diverse sources, including dogs, cats, humans, and environmental samples from veterinary clinics.

We identified an ST551 isolate from a 79-year-old man with frequent contact with his son’s dog. This strain showed an MDR MRSP profile, resistant to 8 of 11 antibiotics tested (Table 2), and containing 10 ARGs, plus mutations in 2 fluoroquinolone resistance genes (Table 2). Comparative analysis with other ST551 isolates from various countries (Supplementary Tables S2 and S3) revealed a consistent resistance pattern, both genotypically and phenotypically. The genetic context of the ARGs (Figure 1) aligns with previous descriptions by Viñes et al. [11], featuring a five-gene cluster (*aph(3′)-IIIa*, *sat4*, *ant(6)-Ia*, *erm(B)*, *dfrG*), and *mecA* within an SCC*mec* type Vc element. Additionally, *tet(K)* was found in a putative plasmid inserted into the chromosome, consistent with previous findings. All ST551 isolates analyzed shared a nearly identical virulence factor profile, with only three genes (*spsD*, *spsI*, and *spsL*) differing (Supplementary Table S4). Isolate 23-150 included these three genes, with SpsD and SpsL being known to play roles in *S. pseudintermedius* infection [49,65]. The consistent ANI values and phylogenetic relatedness (Figure 3) suggest the ST551 lineage is clonal and has spread across Europe with minor genetic changes.

Two of the *S. pseudintermedius* isolates (23-298 and 23-583) were recovered from peritoneal dialysis pericatheter infection and colonization in two young women (33 and 36 years old, respectively). Both isolates belonged to ST2672, exhibiting identical antibiotic resistance patterns and virulence factor profiles. Notably, both patients owned companion animals, specifically two dogs and a cat, respectively. The association between *S. pseudintermedius* infections and catheters has been previously documented. In 2010, Chuang et al. [66] described a catheter-related bacteremia in a 6-year-old boy, initially misidentified as *S. intermedius* but later confirmed as *S. pseudintermedius* through 16S rRNA analysis. The boy had been exposed to a companion dog. In 2019, Diaz et al. [67] reported a catheter-related bloodstream infection in a 36-year-old woman undergoing hemodialysis who had one cat and four dogs. A subsequent case study by Nomoto et al. [68] detailed a 41-year-old man with a central parenteral nutrition system featuring a totally implantable venous access port (TIVAP). Despite being advised to avoid contact with his pet dog, the patient experienced a reinfection 52 days after discharge, with the strain matching the initial one. After avoiding contact with the dog, no further reinfection occurred.

Hence, the potential acquisition of an identical ST2672 *S. pseudintermedius* clone by both patients from their respective companion animals remains plausible. Bierowiec et al. [63] reported a 22.86% MRSP prevalence in cats, Papić et al. [15] identified one ST71 MRSP strain from a cat’s wound, Van Duijkeren et al. [69] isolated MRSP strains from cystitis and otitis externa in two cats, and Abraham et al. [70] reported methicillin-resistant *S. intermedius* (presumably *S. pseudintermedius*) in a cat in 2007. However, the concurrence of the same clone in unrelated individuals suggests a potential nosocomial infection source within the dialysis unit, rather than two separate community-acquired infections through animal contact. To elucidate the origin of the strain, comprehensive investigations, including environmental samples from the hospital and patients’ pets, are needed. Such inquiries are essential to determine whether the hospital environment serves as a reservoir for *S. pseudintermedius*.

All isolates examined in this study harbored several virulence factor genes crucial for *S. pseudintermedius* pathogenesis, including colonization, biofilm formation, and induction of host inflammatory reactions. As Kmieciak et al. noted [71], virulence factors such as the elastin-binding protein Ebps (*ebpS* gene), fibrinogen/fibronectin binding protein SpsE (*spsE* gene), and the exfoliative toxin SIET (*siet* gene) contribute to superficial infections. Toxins like leukocidin (*lukF*-I and *lukS*-I genes) can damage host defense cells and erythrocyte membranes, compromising function and aiding colonization spread [72].

Genes critical for effective colonization, particularly in catheter-related infections, include those mediating biofilm formation. All four isolates harbored the *icaABCD* operon and the *psmαβδε* genes. The *icaABCD* operon is involved in synthesizing the polysaccharide intercellular adhesin, while the *psmαβδε* genes produce modulins that damage host cells and stabilize biofilms. Pompilio et al. [73] found that *S. pseudintermedius* strains associated with human wounds can generate ultrastructurally complex biofilms, providing a protective environment against antibiotics and enhancing pathogenic potential. This underscores the ability of these strains to cause implant-associated infections, such as the catheter-related infections that represented 50% of the cases in this study, posing a risk of life-threatening complications.

To prevent or reduce zoonotic transmissions of *S. pseudintermedius* from dogs to humans, pet owners should be aware of behaviors that could potentially lead to transmission, including feeding dogs in the kitchen, allowing dogs to sleep in the kitchen or on the sofa, and letting dogs lick their faces and/or hands [74], as hands seem to play an important role in transmission via contaminated surfaces [75]. Additionally, *S. pseudintermedius* can persist in the environment for up to 10 weeks [18]. Regarding the issue of MDR isolates, better antibiotic treatment practices should be considered. Mandatory susceptibility testing has been shown to reduce antimicrobial resistance, as does avoiding the misuse of antibiotics through empirical treatment rather than treatment based on susceptibility testing [24,76,77].

## 5. Conclusions

In conclusion, this study documents four cases of human *S. pseudintermedius* infection or colonization in individuals in contact with companion animals. Two clonally related isolates were recovered from unrelated women with peritoneal dialysis catheters, and another isolate belonged to the increasingly prevalent ST551 MLST in Europe. Three out of the four isolates exhibited a MDR genotype and phenotype, and all demonstrated a diverse array of virulence factors linked to the microorganism’s pathogenicity. The scarcity of reported *S. pseudintermedius* human cases, particularly in Spain, underscores the need for more studies on its role in zoonotic infections. Given the widespread ownership of dogs, natural reservoirs of this microorganism, the resistance of different *S. pseudintermedius* lineages to multiple antibiotics poses a potential threat to infection treatment. To determine if the hospital environment acts as a reservoir for *S. pseudintermedius*, thorough investigations, including environmental samples from the hospital and patients’ pets, are needed.

## Figures and Tables

**Table 1:** Information of the host and isolates included in this study. These are all the *S. pseudintermedius* collected in daily routine in the Microbiology department of the Hospital Clínic de Barcelona from February to June (both included) of 2023.

	Patient 1	Patient 2	Patient 3	Patient 4
**Strain Code**	**23-150**	**23-298**	**23-472**	**23-583**
**Date of isolation**	13 February 2023	4 April 2023	5 April 2023	8 June 2023
**Sex**	Male	Female	Male	Female
**Age**	79	33	72	36
**Comorbidity**	Hypertension, left mandibular squamous cell carcinoma	Nephropathic cystinosis, chronic kidney disease on peritoneal dialysis	Diabetes mellitus, coronary artery disease, triple bypass surgery	Hypertension, IgA vasculitis with chronic kidney disease on peritoneal dialysis
**Infection foci**	Infection associated with osteosynthesis plaque in zygomatic arch with bone sequestration	Peritoneal dialysis peri-catheter infection	Surgical wound infection (sternotomy)	Peritoneal dialysis peri-catheter colonization
**Treatment**	Linezolid/Tedizolid, 45 days	Linezolid, 7 days	Clindamycin, 7 days	No
**Outcome**	Cured	Cured, but relapsed later	Cured	-
**Owns a dog?**	No, but regular contact with son’s dog	Two	Two	No
**Owns a cat?**	No	Feeds a stray cat	One	One
**MRSP/MSSP**	MRSP	MRSP	MSSP	MRSP
**MLST**	ST551	ST2672	ST2673	ST2672

MLST, multi-locus sequence type; MRSP, methicillin-resistant *S. pseudintermedius*; MSSP, methicillin-susceptible *S. pseudintermedius*.

**Table 2:** Minimum inhibitory concentrations (MICs, µg/mL) and antibiotic resistance genotype. All the strains that presented the *blaZ* gene also presented the *blaI* and *blaR1* genes from the same operon. Shaded boxes denote resistance in the case of antibiotics, and presence in the case of antibiotic resistance genes.

	Isolate	23-298	23-472	23-583	23-150
	**MLST**	ST2672	ST2673	ST2672	ST551
**Drug class**	**Sçc*mec* type**	ΨSCC*mec*_57395_	-	ΨSCC*mec*_57395_	Vc(5C2&5)
β-lactam	OXA	>2	≤0.25	>2	>2
	*mecA*				
	*blaZ*				
Tetracycline	TET	>2	≤0.5	>2	>2
	*tet(K)*				
	*tet(L)*				
	*tet(M)*				
Aminoglycoside	GEN	≤1	≤1	≤1	>4
	*aac(6′)-Ie-aph(2″)-Ia*				
	*aph(3′)-IIIa*				
	*ant(6)-Ia*				
Fluoroquinolone	LEV	>4	≤0.5	>4	>4
	*gyrA* S84L				
	*gyrA* E871K				
	*parC* S80I				
Macrolide & Lincosamide	ERY	>4	≤0.25	>4	>4
	CLI	>1	≤0.25	>1	>1
	*erm(B)*				
Trimethoprim/sulfamethoxazole	SXT	>4/76	≤0.5/9.5	>4/76	>4/76
	*dfrG*				
Oxazolidinone	LIN	1	1	1	1
Streptothricin	*sat4*				
Glycopeptide	VAN	≤0.5	≤0.5	≤0.5	≤0.5
Rifamycin	RIF	≤0.25	≤0.25	≤0.25	≤0.25

OXA, oxacillin; ERY, erythromycin; CLI, clindamycin; GEN, gentamicin; VAN, vancomycin; RIF, rifampicin; LEV, levofloxacin; LIN, linezolid; SXT, trimethoprim-sulfamethoxazole; TET, tetracycline.

## Data Availability

The genomic data generated in this study, comprising Nanopore fastQ read files and genome assemblies, has been deposited at the National Center for Biotechnology Information (NCBI) under the BioProject PRJNA1043044. Genome accession are as follow: 23-150 JAXBCY000000000, 23-298 CP139908, 23-472 CP139907, and 23-583 CP139906.

## Supplementary Materials

The following supporting information can be downloaded at: https://www.mdpi.com/article/10.3390/microorganisms12081695/s1, Table S1: *S. pseudintermedius* genomes downloaded from NCBI Nucleotide database; Table S2: Supplementary ST551 Minimum inhibitory concentrations (MICs, µg/mL); Table S3: Supplementary ST551 antibiotic resistance genotype; Table S4: Virulence factor content. Genomes are sorted by sequence type.
